# Effects of gender, ejection fraction and weight on cardiac force development in patients undergoing cardiac surgery – an experimental examination

**DOI:** 10.1186/1749-8090-8-214

**Published:** 2013-11-18

**Authors:** Constanze Bening, Helge Weiler, Christian-Friedrich Vahl

**Affiliations:** 1 Department of Cardiothoracic and Vascular Surgery, Medical Centre of the Johannes-Gutenberg-University Mainz, Langenbeckstr.1, 55131 Mainz, Germany

**Keywords:** Calcium sensitivity, pCa, Force relationship, Skinned fibers

## Abstract

**Background:**

It has long been recognized that differences exist between men and women in the impact of risc factors, symptoms, development and outcome of special diseases like the cardiovascular disease. Gender determines the cardiac baseline parameters like the number of cardiac myocyte, size and demand and may suggest differences in myofilament function among genders, which might be pronounced under pathological conditions. Does gender impact and maybe impair the contractile apparatus? Are the differences more prominent when other factors like weight, age, ejection fraction are added?

Therefore we performed a study on 36 patients (21 male, 15 female) undergoing aortic valve replacement (AVR) or aortocoronary bypass operation (CABG) to examine the influence of gender, ejection fraction, surgical procedure and body mass index (BMI) on cardiac force development.

**Methods:**

Tissue was obtained from the right auricle and was stored in a special solution to prevent any stretching of the fibers. We used the skinned muscle fiber model and single muscle stripes, which were mounted on the “muscle machine” and exposed to a gradual increase of calcium concentration calculated by an attached computer program.

**Results:**

1.) In general female fibers show more force than male fibers: 3.9 mN vs. 2.0 mN (p = 0.03) 2.) Female fibers undergoing AVR achieved more force than those undergoing CABG operation: 5.7 mN vs. 2.8 mN (p = 0.02) as well as male fibers with AVR showed more force values compared to those undergoing CABG: 2.0 mN vs. 0.5 mN (p = 0.01). 3.) Male and female fibers of patients with EF > 55% developed significantly more force than from those with less ejection fraction than 30%: p = 0.002 for the male fibers (1.6 vs. 2.8 mN) and p = 0.04 for the female fibers (5.7 vs. 2.8 mN). 4.) Patients with a BMI between 18 till 25 develop significant more force than those with a BMI > 30: Females 5.1 vs. 2.6 mN; p 0.03, Males 3.8 vs. 0.8 mN; p 0.04).

**Conclusion:**

Our data suggest that female patients undergoing AVR or CABG develop significantly more force than male fibers. Additionally we could image the clinical impression of negative impact of overweight and obesity as well as low ejection fraction on cardiac function on level of the myofilaments and observed a reduced force capacity, which is more prominent in male fibers.

## Background

The growing perception of differences among genders regarding development and outcome of CVD and cardiac disease in general has been explored in cellular, molecular and genetic levels. Gender determines the cardiac baseline parameters like the number of cardiac myocyte, size and demand and may suggest differences in myofilament function among genders. Gender plays also an important role in the detrimental effects of the aging process [1] and we recognize increasingly that cardiovascular disease manifests itself differently in women and men, for example more women than men with cardiovascular disease present with preserved and not impaired LV systolic function [2]. And suggesting that death risk increases with worsening left ventricular systolic LV function, the data are also less conclusive than expected. Konhilas assumes that the increased survival of women is due to the elevated contractile function, but other studies [3,4] argue that there is a lack of survival benefit for patients with preserved LV function as measured by ejection fraction to those with impaired LV function. Hsich [5] assumes that there must be a fundamental difference in the ability of the female heart to tolerate physiological changes associated with cardiac heart failure. To evaluate the underlying mechanisms many studies have been performed:

Some studies [6-9] suggest differences in excitation and contraction coupling cycle, which are gender-related. Possible explanations for these observations are differences in calcium release from sarcoplasmatic reticulum [7,8] by genetically determined expression of calcium sensitive proteins [10,11]. Different experimental studies [2] have suggested a fundamental association between myofilament Ca2 + -sensitivity, sarcomere length and tension of the cardiac cell as well as pressure and volume in the intact ventricle. But whereas some studies in female rat hearts demonstrate an increased calcium sensitivity than male counterparts, [3,12-14], other studies show the opposite result [15,16]. So the literature presents itself inconsistently.

So in summary gender seems to determine the contractile fate of the myofilaments under healthy and pathological conditions.

Therefore we performed a study with human tissue and examined the influence of gender on the contractile performance, the correlation to the ejection fraction and body mass index.

## Methods

### Origin of samples

The tissue was taken from the right auricle before patients were connected with the extracorporal circulation. The right auricle was taken from 36 patients (21 male, 15 female), undergoing cardiac surgery. All patients were informed and gave written consent to use the tissue and the approval from the ethic committee (Ärztekammer Rheinland Pfalz, Ethikkommission) is available for this study. The tissue was immediately stored and transported in an oxygenated cardioplegic solution. All patients gave written informed consent before surgery. Patients with terminal congestive heartfailure, inflammatory heart disease, re-do procedures and chronic defibrillation were excluded from the study.

### Patient’s clinical characteristics

We examined tissue from 36 patients: 21 male and 15 female patients. The mean age of the male patients was 61 years and in the female group 68 years (see Table 1). 21 Patients underwent aortic valve replacement and 15 underwent CABG operation. 24 patients had an EF of more than 55%. Three patients had an EF between 45-50%, another three patients had an EF of 40-45% and 35-40%. One patient had an EF of 35% and two patients even less than 30%. 15 patients had an BMI between 18 and 25, 17 patient’s BMI was between 25 and 30 and four patients showed an BMI more than 30. 32 patients had arterial hypertension and 22 patients had diabetes. 28 patients took medication like beta blockers, ACE inhibitors and anticoagulation like aspirin. Eight patients had paroxysmal atrial fibrillation and needed vitamin k antagonists.

**Table 1:** Overview of patient’s data

		**Male**	**Female**
Sample size		21	15
Mean Age (years)		61	68
AVR		8	10
CABG		13	5
Mean BMI			
	CABG	25	27
	AVR	25	24
Mean EF			
	CABG	67%	47%
	AVR	54%	51%

### Tissue preparation and experimental setup

To protect the tissue, we transported the tissue in an oxygenated cardioplegic solution, using BDM (Butanedione-Monoxim, 30 mM) as ATP-sensitive potassium canal inhibitor. We performed the skinning procedure in a special solution (Contents [mM]: Imidazol 68,08; Sodium azide 65,01; Ethyleneglycol tetraacetic acid 380,4; Dithioerythriol 154,3; Magnesium chloride 203,3; ATP 605,2). After resecting the trabecular out of the auricle, we stored the tabecula in a solution with the same content, containing 1% Triton-X-100 in addition, for 24 hours at 4°C.

After that time we took the trabecula out of the solution and prepared them for the experimental set up. The fibers need to be prepared for the muscle machine i.e. they are cut in stripes with a size of 2-2,5 mm × 0,3 mm. We then immersed the fibers in a solution called relaxation solution (Imidazol 68,08, Creatinphosphat 327,2; Sodium azide 65,01; EGTA 380,4; Magnesium chloride 203,3; Dithioerythriol 154,2; ATP 605,2) to prestretch them and afterwards they will be exposed to a contraction solution, containing calcium (same content like the relaxation solution except for calcium chloride).

The desired calcium concentrations were calculated by a computer program, following the equation of Fabiato & Fabiato and given as pCa (-log of free [Ca]_+2_).

The fibers are exposed to a continuous increase of calcium concentration, starting at pCa 8.0 and ending at pCa 4.5. The rising calcium concentration and force development were simultaneously recorded and sampled on an IBM-compatible computer. The experimental setup and computer controlled feedback circuit were purchased from Scientific instruments, Heidelberg, Germany.

### Statistical analysis

We performed an experimental cycle with 5 steps of calcium concentration. We took three fibers from each patient undergoing the cycle of increasing calcium concentration. We tested the Gausian distribution with the Shaphiro-Wilk Test. Afterwards we used the T.test for independent values and a significance level of 5% was kept. The influence of the different factors was tested with Anova method.

## Results

**Figure 1:**
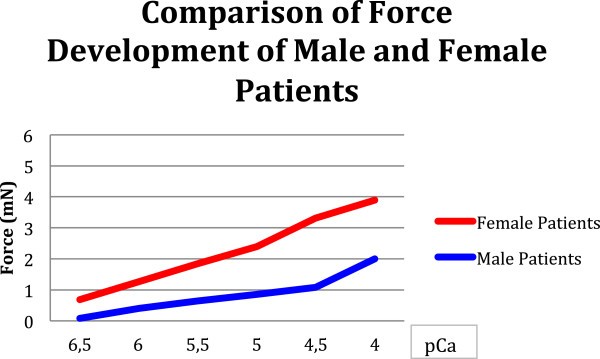
Male and female patients develop significantly different force values (p 0.03).

The 21 male fibers developed 2.0 ± 0.2 mN at the highest step of calcium concentration, whereas the female fibers achieved 3.9 ± 1.2 mN (see Figure 1). The mean half maximal activation was achieved at pCa 5.5 in the male group and at pCa 5.0 in the female group. The greatest increase in force development is achieved at the lowest step of pCa 6.5 to pCa 6.0 in both genders, with almost three-fold force values.

**Figure 2:**
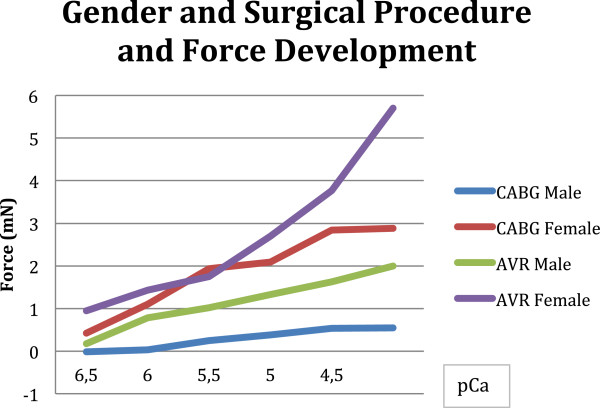
The difference of gender related force development can be underlined when focused on the surgical procedure (Females CABG vs. AVR p 0.02; Males CABG vs. AVR p 0.01).

This difference is even more pronounced when focused on surgical procedure (see Figure 2): female fibers undergoing aortic valve replacement achieve 5.7 mN whereas the male patients do not develop more than 2.0 mN at pCa 4.0, although the male fibers had a mean EF of 54%. This difference is significant (p = 0.01). The mean EF of the female fibers was 51%. Assuming that maybe patients with a preserved ejection fraction are included in this group and impaired diastolic function, we found in 2 female patients of the AVR group a diastolic dysfunction grade I and in one male fiber a diastolic dysfunction grade II. The pCa_50++_ for the female fibers is achieved at pCa 5.0, for the male fibers at pCa 5.5 (see Table 2).

**Table 2:** Overview of significant and not significant results

		**Male**	**Female**	**Among genders**
Age				
	<40 years	0.07	0.09	
	40-60 years	0.04	0.05	
	60-80 years	0.001	0.02	
EF in relationship to EF > 55%				
	< 30%	0.002	0.04	
	30-35%	n.s.	n.s.	
	35-40%	n.s.	n.s.	
	40-45%	n.s.	n.s.	
	45-50%	n.s.	n.s.	
BMI in relationship to BMI > 30				
	18-25	0.04	0.03	
	25-30	n.s.	n.s.	
Force development and age				
(Pmax/40-60y)		0.02	0.01	0.01
	BMI > 25	0.002	0.02	0.01
	BMI < 25	0.001	0.003	
Cardiac diagnosis and mean Age (years)				
	CABG		75	80
	AVR	45	63	
Surg. Proc				
(AVR vs. CABG)		0.01	0.02	

A similar impression can be seen in the CABG group, the male fibers achieve 0.5 mN and the female fibers 2.8 mN. The biggest increase in force is found again in the lowest step of calcium concentration (from pCa 6.5 to 6.0). The half maximal activation for the males is achieved at pCa 5.0 and for women at pCa 6.0. When compared to the patients undergoing valve surgery we see a difference in male as well as in female patients: p = 0.02 for the females (AVR versus CABG) and p = 0.01 for the males (AVR versus CABG).

We furthermore performed an analysis about potential differences in the distribution of cardiac diagnosis, ejection fraction and BMI in men and women:

Five female patients underwent aortocoronary bypass operation and 10 underwent aortic valve replacement. 13 male patients had also aortocoronary bypass operation and 8 underwent aortic valve replacement. All patients undergoing aortocoronary bypass operation had a 3-vessel-disease and therefore needed both internal mammary arteries and additionally the vena saphena magna for revascularisation. All valve patients underwent aortic valve replacement with a superior ministernotomy, as routinely used in our department. In the female group, the mean EF (%) in the CABG group was 47%, in the AVR group 51%. The mean EF in the male CABG group was 67% and in the AVR group 54%. The mean BMI in the male group was 26 and in the female group 25. Regarding the operation procedure the BMI averaged 25 in the male CABG group and also 25 in the male AVR group. In the female CABG group the mean BMI was 27 and 24 in the AVR group.

To keep a close eye on the ejection fraction we divided the groups into:

➢ >55%

➢ 45-50%

➢ 40-45%

➢ 35-40%

➢ 30-35%

➢ < 30%

**Figure 3:**
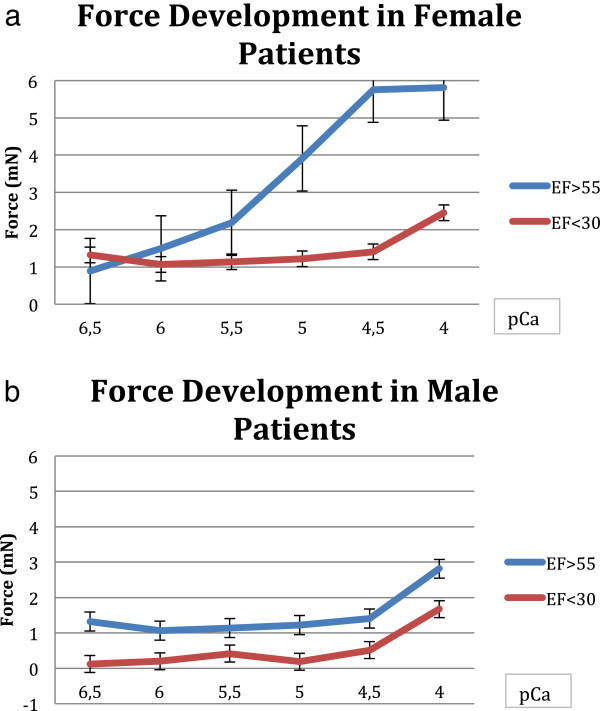
**Force development and ejection fraction in male female patients. a**. Dependency of force values on ejection fraction (EF > 55% vs. EF < 30%) in female patients (p 0.04). **b**. Dependency of force values on ejection fraction (EF > 55% vs. EF < 30%) in male patients (p 0.002).

The male fibers with an EF > 55% differ significant from the fibers with less than 30% EF, this was significant (p = 0.002) It is interesting to see, that the male fibers differ in all groups from those with an EF > 55%: p = 0.003 (EF 45-50%), p = 0.003 (EF 40-45%), p = 0.009 (EF 35-40%), p = 0.06 (EF: 30-35%). This is different to the female fibers: only one group behaves significant differently from those patients with more than 55% EF: the patients with an EF less than 30% (p = 0.04). All other groups are not significantly different (see Figure 3a and b).

We furthermore evaluated if there might be a difference of distribution of gender, cardiac diagnosis and BMI in the patients with impaired and normal EF and examined the groups as follows:

There were two female patients with an EF lower than 30%, both underwent aortic valve replacement. The BMI in this group was 27. The group with an EF 30-35% consisted of one male patient, undergoing CABG with a mean BMI of 24. In the group with an EF between 35-40% were two female patients and on male patient: the male patient and one female patient underwent CAGB and the other female had an AVR operation. The mean BMI was 28. The next group with an EF between 40-45% presented only male patients, undergoing one CABG and two AVR operations with a mean BMI of 32. In the group with an EF between 45-50% we found 1 female and 2 male patients. The female patient and one male patient had an AVR, the other patients had CABG procedure, mean BMI was 24. The last group with patients with an EF over 55% consisted of 6 female patients undergoing AVR and 4 patients undergoing CABG. Additionally there were 10 male patients undergoing CABG and 4 patients undergoing AVR. The mean BMI in the female group was 25 and in the male group 27.

Refering to the weight, we also divided the patients in groups after BMI:

➢ BMI 18-25

➢ BMI 25-30

➢ BMI > 30

The male fibers with an BMI of more than 30 showed obvious less force values than those patients with an BMI between 18-25: 0.8 mN versus 3.8 mN, whereas no differences could be seen between the force values of patients with BMI between 18-25 and those between 25 and 30. This is similar to the female patients: The female fibers develop significantly less force with a BMI > 30 compared to those with a BMI between 18-25: 2.6 mN versus 5.1 mN. But also the female fibers, having a BMI between 25-30 did not show significant differences to those with a BMI between 18-25. Additionally the ejection fraction of the patients with a BMI over 30 was 40%, whereas the patients with a BMI between 18-25 had an EF of 50% and the patients in between had an EF of 55%. Also in this group a potential different distribution of gender, diagnosis and EF was tested in the patients with high or low BMI:

Defining a high BMI as a BMI > 25 and a low BMI with a BMI < 25, we found the following distribution: In the female group 8 patients had a BMI higher than 25 and 7 patients had an BMI lower than 25. The male group presented 13 patients with a high BMI and 8 with a low BMI. Regarding the diagnosis we found in the CABG group 8 male patients and 3 female patients with a high BMI, whereas 5 male patients had a low BMI in the CABG group and 2 female patients with a low BMI in this group. In the AVR group we found 5 male and 5 female patients with a high BMI, and 3 male and 5 female patients with a low BMI. The male patients with a BMI less than 25 and with AVR operation procedure had a mean EF of 58%, those undergoing CABG had a mean EF of 56%. In the high BMI group the male patients undergoing AVR had a mean EF of 42% and those with a CABG procedure had a mean EF of 49%. In the female group we found a mean EF of 50% in the low BMI group undergoing AVR. In the high BMI group the mean BMI was 48% for AVR procedure and 47% in the CABG group.

In a final step we evaluated a possible impact of age on the cardiac force development and a potential correlation with the other factors:

We examined the influence of age on cardiac force, cardiac diagnosis, BMI and EF and tested for statistical significance (see Table 2):

The impact of age on the force values, achieved at the highest calcium concentration (pCa 4.0), was significant in men and women at higher ages: At the age between 60-80 we found significant correlations between age and the Pmax (female 0.01, and male 0.02) and also among gender: p:0.01.

Comparing the age to the EF we also found some evidence for significant influence: When we tested the age with the described EF we found significant p values for both genders in almost all groups: < 40 years: male p 0.07; female 0.09; 40-60 years male 0.04; female 0.05; 60-80 years: male 0.001; female 0.02.

When we tested a possible correlation of age and BMI we found some evidence for significant results both in the male group with an BMI less (p 0.002) and more than 25 (p 0.001) and in the female group with an BMI less (p 0.003) and more than 25 (p 0.01) as well as among genders (p 0.004).

Referring to the cardiac diagnosis we saw that female patients undergoing CABG were older (Mean age 80 years) than male patients with 75 years, whereas male patients undergoing AVR were significant younger (Mean age 45 years) than female patients (63 years). This difference was significant among genders p 0.02.

## Discussion

We observed clear differences in cardiac force development between female and male patients in general: Women seem to produce higher force values compared to men, but male fibers had a higher sensitivity to calcium and pCa 50 was achieved at pCa step 5.5 whereas female fibers need more calcium to achieve half maximal activation. This is in accordance to the findings in literature, that female fibers develop more force to positive inotropic activation [14] and male fibers need significant less calcium for pCa_50++_ compared to females [15]. Regarding to calcium sensitivity, the male fibers show a higher sensitivity (pCa 5.5), whereas female fibers achieve half maximal activation at higher steps of calcium concentration. These observations are similar to those found in rats [2], that there is reduced calcium sensitivity in the hearts of females compared to male. The finding can support the observation, that the loss of estrogen after ovariectomy increases sensitivity of myofilaments to calcium [17-19]. It is furthermore interesting to see that loss of testosterone in males reduced calcium sensitivity, which can be reversed by testosterone replacement [20]. As mentioned, the results in literature also show the opposite and some studies even show no significant difference among genders [12-14]. The reasons for that are multifold: first of all the experimental approaches are different. In our case we performed the study with skinned fibers, which means that all membrane-dependent processes are excluded. For example the sarcoplasmatic reticulum is removed, which is often mentioned to be one reason for the sex differences. Furthermore the described differences in the excitation-contraction coupling still remain intact in nonpermeabilized preparations. And additionally the calcium sensitivity is species dependent [21-24]. A potential correlation of sex, cardiac disease and disease progression obviously exists, but it is still not known how sex influences the signaling pathways, which impacts the contractile properties [2].

It is furthermore interesting to see, that the impact of weight can be found in the force values. There are some evidences that the adipose tissue is a highly endocrine organ and that this tissue exerts highly potent cardiodepressant activity with a direct effect on cardiomyocytes contraction [8], which might support our findings of reduced force values associated with high BMI.

The male patients with a BMI > 25 had an EF of 42% (AVR) and 49% (CABG), this can also be seen in the female group (AVR 48%, CABG 47%). In the male group with a normal BMI (i.e. BMI < 25) the EF was 58% AVR) and 56% (CABG), similar to the females (50% AVR). This in accordance to different studies, who can all show a significant decrease of contractility parameters associated with obesity, for example in isolated perfused rat hearts [25-27]. All contractility parameters as well as coronary flow significantly decrease within a few seconds of incubation with adipocyte factors [26,28]. We observed significant lower force values in male and female patients with a BMI between 18-25 and those with a BMI over 30. The group in between did not show significant differences in the force values compared to the both other groups. This is a unique observation in literature: obesity is associated with impaired LV relaxation and diastolic calcium overload and leads to prolonged interaction of myofilaments [25]. Furthermore the diastolic dysfunction aggravates and proceeds towards systolic dysfunction. Some studies actually talk about obesity cardiomyopathy because of the structural and functional alterations [25]. We can support these studies with the significant reduced force values we achieved in our measurements. Additionally the patients with a BMI > 30 had a mean ejection fraction of 40%, whereas the patients with a BMI between 18-25 had an EF of 50%. So we can assume, that patients with a high BMI (male as well as female) had a lower EF than those with normal BMI. This was similar in both genders. So the clinical observation of a reduced left ventricular function could be imaged by the force values, we measured.

When we also add the age and cardiac diagnosis, we found significant influence for both factors: And female patients with coronary heart disease requiring CABG were significant older than female patients with an aortic stenosis undergoing AVR (80 years vs. 63 years), this similar to the males (CABG: 75 years, AVR: 45 years.). Considering a potential correlation of force values and cardiac diagnosis, we observed also clear differences: women undergoing aortic valve replacement achieve the highest force values, although both genders are described to have a “normal” ejection fraction, but male patients requiring CABG procedure show significant less force and EF values [29]. Aurigemma made an interesting observation [30], which can support our measured values: elderly women with higher degree aortic stenosis show more marked concentric hypertrophy, lower levels of wall stress, but higher indices of systolic function than males. A similar observation was made by Weinberg [31]: female rats with aortic stenosis showed 6 weeks after the intervention similar left ventricular hypertrophy and systolic wall stress but preserved contractile reserve. Montalvo [32] gives an explanation in her study by suggesting that circulating androgens involve TGF-ß and cause thereby the detrimental effects in the myocardium. Refering to the results in the CABG group, the results are even more pronounced. Male fibers develop even less force than in the AVR group. This could be explained with the observed cellular changes in patients with myocardial ischemia, undergoing CABG procedure and literature gives supporting evidences: Fang [33] found in male rats with myocardial infarction a greater extent of LV remodeling and a more reduced muscle strength compared to female rats. Furthermore signs of inflammatory response were increased in the male rats after myocardial infarction [33]. Bhupathy [1] furthermore showed in her study about heart disease in men and women, that changes in contractility are associated with alterations in the expression of key calcium handling proteins and that the levels of these proteins are consistently higher in females than in males. She suggests, that the different expression of these proteins may play a role in how the heart responds to cardiac dysfunction [34]. The same observation can be made when we take cardiac fibrosis as the end stage of pressure and volume overload: several studies show that male rats with HF show more severe fibrosis. [31]. This might be an explanation for the high difference of force values of males undergoing CABG procedure and females having AVR.

The significant impact of age on the contractile function is not a surprising issue, but however the processes how age can impair myocardial function is not well understood. Howlett performed a study about age related changes in excitation-contraction coupling, which are more prominent in ventricular myocytes from male rats than those from females rats [7] and gives additional age related changes of cardiac contraction [9]: for example the ability to increase contraction amplitude in response to catecholamines or increased stimulation frequency is impaired in aging myocytes, furthermore the calcium transients, needed for the calcium-induced Ca^2+^ release, are disrupted in aging myocytes. There are also some evidences in literature, that age-related changes in the heart might be more prominent in men than women [6,29].

Refering to the issue of transferability of observations derived from tissue, which was harvested from the right atrium of patients, to a cardiac pathology, which mainly affects the left heart side, is of course difficult but quite possible. Vannier examined the specifities of myofibrils in cardiac muscle from atria and ventricle and made an interesting finding: the adult atrial tissue shows the same contractile properties than ventricular tissue, but differs mainly in metabolic properties [23]. In opposite to that, Wankerl examined [18] the calcium sensitivity in various kinds of cardiac disease and found out, that the calcium sensitivity from ventricle was higher in all patients at about 0.14pCa Units, but was the same among the different heart diseases, whereas atrial fibers differed among the different cardiac diseases. This is probably an evidence for the assumption that cardiac pathologies can be better imaged in atrial tissue than in ventricle tissue maybe because of adaption to elevated pressure conditions of the ventricular fibers. Considering the different pressure and volume relations in the human ventricle and atrium, it is assumable, that calcium sensitivity and contractile proteins may differ. But nevertheless cardiac pathology in the different diseases will not exclusively affect the left heart side and differences of calcium sensitivity of atrial tissue in various cardiac diseases are already described [18] and might allow a representative statement to the contractile behavior.

In summary we observed a significant influence of gender on cardiac force development which suggests a reduced force capacity of male fibers. We furthermore observed a significant influence of cardiac diagnosis (coronary heart disease requiring CABG procedure or aortic stenosis requiring AVR): both genders with coronary heart disease develop lower force values than those patients with an aortic stenosis. We found a significant influence of weight, which was significant when compared patients with an BMI < 30 and those with a BMI between 18-25 and we found a significant correlation of age and force development: the older the patients are the lower are the forces, but this difference was more prominent in males: they develop at higher age even lower force values than the females. This aspect can also be seen when we compare age and EF: The EF is reduced in higher steps of age and always more pronounced in males.

## Conclusion

We conclude, that cardiac function is associated with a complex correlation of age, gender, weight, ejection fraction and cardiac diagnosis. Considering that the impact of gender is already controversially discussed in literature it seems more comprehensive that the discussion of influence becomes more and more complex and difficult when including all other factors. It is not possible to isolate the influence if the single factors. But it seems acceptable that the cardiac contraction is an equation with many different variables.

Clinical perspective: Considering the increasing body of studies about “gender medicine” and the issue of gender related risc factors and associated morbidity and mortality the main clinical interest might be a preventive one: reducing risc factors like body weight, starting the check-ups at lower age for males, early indicate therapy in patients undergoing CABG. As a future aspect it might be possible to create an individual medical profile for example in calcium sensitivity and force capacity and provide special inotropic therapy (Inodilators) before and after cardiac surgery to reduce the impairment of cardiac contractile function by additional factors like extracorporal circulation, cardioplegic solution. This aims at an individualized medicine which recognized each patient as a special medical challenge.

Limitation of the study: It is difficult to talk about statistical significance in a limited number of samples. In this study it is additional difficult to differentiate the influence of the single issue like age, gender, weight, ejection fraction because all factors are dependent and influence themselves. Beside technical limitation, like the handling of every skinned fiber, the accuracy of the experimental set up, we need to enlarge the sample size to evaluate the statistical significance.

## Abbreviations

ATP: Adenosintriphosphat; AVR: Aortic valve replacement; BDM: Butanedione-Monoxim; BMI: Body mass index; CABG: Coronary artery bypass grafting; CVD: Cardiovascular disease; EF: Ejection fraction; LV: Left ventricle; pCa: Calcium concentration; pCa50**: Calcium sensitivity.

## Competing interests

The authors declare that they have no competing interests.

## Authors’ contributions

CB: Acquisition of tissue, preparing the tissue, carrying out the experiments, data analysis, drafting the paper. HW: Involved in drafting the paper and interpretation of data. CFV: Revision of the paper, contributed to the conception of the study. All authors read and approved the final manuscript.
